# Scrotal pain as a novel symptom of multisystem inflammatory syndrome in children (MIS-C): Report from Riyadh, Saudi Arabia

**DOI:** 10.12669/pjms.39.3.6257

**Published:** 2023

**Authors:** Lama AlFakhri, Abdullah F. AlSaeed, Majed B AlZin, Syed Amir Ahmad

**Affiliations:** 1 Dr. Lama AlFakhri, MD Department of Emergency Medicine, King Khalid University Hospital, King Saud University Medical City, King Saud University, Riyadh, Saudi Arabia; 2 Dr. Abdullah F. AlSaeed, Medical Student, King Khalid University Hospital, King Saud University Medical City, King Saud University, Riyadh, Saudi Arabia; 3 Dr. Majed B AlZin, MBBS, King Khalid University Hospital, King Saud University Medical City, King Saud University, Riyadh, Saudi Arabia; 4 Dr. Syed Amir Ahmad, MD (Peds), MRCPCH (UK), Department of Emergency Medicine, Ped Emer Fellowship (University of Toronto), King Khalid University Hospital, King Saud University Medical City, King Saud University, Riyadh, Saudi Arabia

**Keywords:** MIS-C, Scrotal pain, Riyadh, COVID-19

## Abstract

A previously healthy child, presented with severe abdominal and scrotal pain with scrotal swelling for five days. There was associated fever, vomiting, and diarrhea. There was history of COVID-19 infection in the previous month. The patient was febrile (39°C), and in pain. His other vitals were unremarkable. Testicular torsion and appendicitis were ruled out by ultrasound. Abdominal CT scan showed signs indicating terminal ileitis. His MIS-C panel revealed elevated inflammatory markers and cardiac enzymes and positive SARS-CoV-2 IgG levels. All cultures and RT-PCR COVID-19 were negative. Echocardiogram showed only minor mitral and tricuspid regurgitation. The patient was diagnosed as a case of MIS-C. and recovered completely on management. Our patient showed an inexplicable previously unreported complaint of scrotal pain and swelling as a symptom of MIS-c. Further research tackling MIS-C’s different presentations and comparing the efficacy of the different treatment methods will help us better manage this disease.

## INTRODUCTION

The novel coronavirus disease 2019 (COVID-19) has emerged as a pandemic since the first case was documented in Wuhan, China, in 2019.^1^ Fortunately, COVID-19 was observed to have a mild course in the pediatric age group. However, there are various reports describing a new sequalae named Multisystem inflammatory syndrome in children (MIS-C) affecting children infected with SARS-CoV-2.

MIS-C is described by the Center for Disease Control and Prevention (CDC) as a Kawasaki-like severe inflammatory syndrome that occurs in children positive for infection of SARS-CoV-2.^2^ It was first observed in the UK in April 2020 in eight healthy children who presented with multiorgan failure. Later, the disease was observed in New York City, followed by other countries.^2^ MIS-C case definition includes individuals aged below 21 years who present with a fever greater than 38°C that lasts 24 hours, have a high index for inflammation, and are severely ill with multisystem organ involvement. They should have no other diagnosis that better explains their symptoms and should have tested positive for SARS-CoV-2 by RT-PCR, serology, or antigen test in the past four weeks.^3^

Children with MIS-C have signs and symptoms of persistent fever, abdominal pain, vomiting, diarrhea, skin rashes, lesions, and in extreme cases, hypotension and shock.^3^ It is a newly recognized entity and not all patients share the same signs and symptoms. MIS-C can have a delayed onset weeks after a child had symptomatic or asymptomatic SARS-CoV-2 infections.^3^

There are no specific diagnostic tests for MIS-C. Inflammatory markers are usually elevated with evidence of organ dysfunction. Supportive management in conjunction with intravenous immunoglobulin (IVIG) and steroids is the primary treatment for MIS-C.

Here, we describe a case of MIS-C who presented with chief complaint of scrotal pain, a previously unreported symptom of the disease, highlighting the need for better diagnostic and treatment guidelines for MIS-C.

### Case Description:

A previously healthy 8-year-old boy presented to the emergency department with five-day history of right lower quadrant abdominal and left sided scrotal pain associated with feeling of scrotal swelling. There was associated fever (reaching 39°C), vomiting of food content, and watery diarrhea. There was a history of COVID-19 infection in the earlier month, but no history of trauma, urinary symptoms, mucocutaneous involvement, conjunctivitis, rashes, or any other associated symptoms.

On physical examination, the patient was alert but febrile, and in pain. His vital signs revealed a blood pressure of 108/68 mmHg, heart rate of 127 beats/min, respiratory rate of 24 breaths/min, temperature of 37.8°C, and SpO_2_ of 96%. Oral examination revealed a mildly inflamed throat. There was tenderness of the right iliac fossa with voluntary guarding and a non-distended abdomen with normal bowel sounds. Genital examination showed severe tenderness of the left scrotum, a preserved cremasteric reflex. Respiratory and cardiovascular examinations were unremarkable. No lymphadenopathy, ulcers, rashes, or joint pain was observed. His pain only partially subsided after hydration and analgesia.

Ultrasonography and computerized tomography (CT) ruled out testicular torsion and appendicitis, respectively. Scrotal ultrasonography revealed no evidence of testicular torsion or epididymo-orchitis. Abdominal CT however showed, a thick-walled terminal ileum with signs of inflammation indicative of terminal ileitis.

As a result, he was admitted to the pediatrics department for further observations and work up as a possible case of MIS-C. His MIS-C panel tests revealed high levels of inflammatory markers (ESR, CRP, LDH, Ferritin), and elevated hepatic and cardiac enzymes (troponin, BNP) (Table-I). Blood, throat, urine, and stool cultures, along with RT-PCR COVID-19 test, returned negative. The SARS-CoV-2 IgG test was however positive, confirming the history of a previous COVID-19 infection. Baseline echocardiogram (ECHO) showed normal coronary arteries, mild mitral and tricuspid regurgitation, and an overall normal cardiac function. In addition, his ECG was normal.

**Table-I:** Laboratory test results.

	Admission	Discharge	1st follow-up	2nd follow-up
WBC (x 10^9^/L)	7.300	10.700	10.500	5.600
Hemoglobin (gm/L)	121	81 (L)	121	123
Platelets (x 10^9^/L)	269.0	591.0 (H)	829.0 (H)	341.1
ESR (mm/hr)	76 (H)	-	-	27 (H)
CRP (mg/L)	257.800	103.000	4.340	-
Ferritin (mcg/L)	264.3	-	-	-
LDH (unit/L)	335 (H)	-	-	-
ALT (unit/L)	57	36	82.0 (H)	50
AST (unit/L)	-	49 (H)	53 (H)	43 (H)
Troponin-I (ng/L)	161.000 (H)	-	15.900	-
BNP (pg/mL)	4,893.00 (H)	1,238.00 (H)	-	-
Fibrinogen (gm/L)	5.85 (H)	-	2.71	-
D-Dimer (mcg/mL)	-	1.89 (H)	0.50 (H)	-
Interleukin-6 (pg/mL)	161.40 (H)	-	-	-

WBC: White blood cells, ESR: Erythrocyte sedimentation rate CRP: C-reactive protein, LDH: Lactate dehydrogenase, ALT: Alanine aminotransferase. AST: Aspartate aminotransferase, BNP: Brain-natriuretic peptide (H) and (L) indicate abnormal high and low value.

The patient received multidisciplinary team care as a case of MIS-C. Rheumatology, cardiology, infectious diseases, and urology units jointly handled the case. The patient was administered Intravenous Immunoglobin (IVIG) 2 g/kg over 12 hours, methylprednisolone IV 2 mg/kg per day twice daily, aspirin 81 mg once per day orally, and esomeprazole 10 mg per day. Ceftriaxone (1 mg/day) was empirically administered for three days.

Urgent urological consultation was performed in the emergency department for the scrotal pain. No identifiable genitourinary source for the pain could be identified. Urology team advised symptomatic management and follow up for the scrotal pain.

After a four day admission course, the patient was deemed fit for discharge on a tapering dose of prednisolone and aspirin 81mg for six weeks. Follow-up visits were organized with rheumatology and cardiology clinics after discharge.

On clinic visits, the patient was reportedly doing well and was returning to his normal activities with no complaints. His medications were discontinued after completion of their course. Follow up ECG remained unremarkable and blood investigations results improved to baseline values.

## DISCUSSION

Our patient presented with a fever, mildly inflamed throat, abdominal pain and inexplicable left scrotal pain. Two case reports we found in the literature discussed similar presentations; epididymo-orchitis in pediatrics with MIS-C. Both had unilateral inguinal swelling and erythema.^4^,^5^ Another reported scrotal ulcers as a presentation of MIS-C.^6^ Yet, these presentation remains rare for MIS-C, which indicates the need to remain vigilant when dealing with its differing presentations. To the best of our knowledge, this is the first reported case of scrotal pain as a symptom of MIS-C in Riyadh, Saudi Arabia. Literature discussed some theories explaining why MIS-C might target testicular cells. One theorized that due to MIS-C sharing its mechanism of cellular invasion with SARS-CoV via Angiotensin Converting Enzyme 2 (ACE2). Due to the plethora of cells with ACE2 expression in the genitourinary tract, testicular cells might be a target for the disease. 5 Nonetheless, no clear mechanism is established yet.

The pathophysiology of MIS-C is still not fully understood, although it shares many symptoms with Kawasaki disease and toxic shock syndrome.^7^,^8^ Kawasaki involves small-to-medium-sized arteries in the body and causes many serious problems, including coronary artery aneurysms, myocardial infarctions, and pericarditis.^9^ MIS-C’s most common symptoms including persistent fever, abdominal pain, vomiting, diarrhea, skin rashes, and lesions, overlap with Kawasaki disease, The most serious complications of MIS-C are myocarditis, cardiac dysfunction^7^, and acute kidney injury.^3^ A multicenter cohort study found that gastrointestinal involvement was present in 78-92% of the patients.^10^ Findings in a case series performed in Saudi Arabia similarly showed dehydration, decreased air entry bilaterally, wheezing, and Kawasaki disease criteria in their patients.^11^

There is no diagnostic test for MIS-C. However, given its association with cardiac disease, hospitals perform cardiac investigations (such as ECHO, electrocardiogram, cardiac enzyme, and BNP) and other organ evaluations to assess disease severity and rule out other diseases.^3^ An array of markers can be seen to be elevated indicating an active inflammatory course and damage to the organ systems.^3^ Our patient’s laboratory results likewise revealed elevated levels of inflammatory markers, cardiac enzymes, and liver enzymes.

Treating MIS-C poses a problem due to the paucity of evidence comparing the efficacy of various treatment options.^3^ Treatment regimens aim to provide supportive care and reduce inflammation. IVIG and steroids decrease inflammatory response. General supportive care includes fluid resuscitation, inotropic and respiratory support, pain and fever management. Aspirin is indicated for preventing thrombo-embolic phenomena.^12^ Our patient was managed on similar lines with supportive care, empiric antibiotic, IVIG, steroids and aspirin. Follow-up outpatient appointments confirmed the resolution of his symptoms.

## CONCLUSION

The diagnosis and management of MIS-C remain a challenge. Our patient had inexplicable complaint of scrotal pain and swelling, which could be interpreted as a new symptom of MIS-C. Further research tackling the presentations of MIS-C and comparing efficacy treatment protocol will help us better manage this disease.

### Authors Contribution

**LAF, AAS, MAZ, SAA** collected information, reviewed literature, drafting of manuscript and are responsible for integrity of research.

**LAF**, **AAS**, **MAZ**, did data collection and manuscript writing

**SAA** conceived case report, reviewed and did final approval of manuscript

## References

[1] Joint WHO-China Study Team (2021). WHO-convened global study of origins of SARS-CoV-2:China Part (Text Extract). Infect Dis Immun.

[2] Multisystem Inflammatory Syndrome in Children (MIS-C) Associated with Coronavirus Disease 2019 (COVID-19) Centre for Disease Control and Prevention Health Alert Network–00432.

[3] Information for healthcare providers about multisystem inflammatory syndrome in children (MIS-C) Centers for Disease Control and Prevention.

[4] Sudeep KC, Muthuvel R, Hussain N, Awasthi P, Angurana SK, Bansal A (2022). Epididymo-Orchitis:A rare manifestation of mis-C. Indian J Pediatr.

[5] Haydar M, Baghdadi S, Taleb M, Al-Dali B, Badr H, Ghanem Y (2021). Orchiepididymitis in the context of multisystem inflammatory syndrome in a child with Covid-19 from Syria:a very rare presentation for SARS-Cov-19 in children. Oxf Med Case Reports.

[6] Khan HQ, Srinivas SM, Sanjeeva GN, Swamynathan S, Shivappa SK (2022). Scrotal ulcers in an infant with multisystem inflammatory syndrome in children associated with severe acute respiratory syndrome coronavirus 2 infection. Pediatr Dermatol.

[7] Saeed S, Rajani R (2021). The cardiovascular complications in COVID-19:Focus on acute cardiac injury. Pak J Med Sci.

[8] Gamez-Gonzalez LB, Moribe-Quintero I, Cisneros-Castolo M, Varela-Ortiz J, Muñoz-Ramírez M, Garrido-García M (2018). Kawasaki disease shock syndrome:Unique and severe subtype of Kawasaki disease. Pediatr Int.

[9] Rowley AH, Shulman ST (2010). Pathogenesis and management of Kawasaki disease. Expert Rev Anti Infect Ther.

[10] Al-Harbi S, Kazzaz YM, Uddin MS, Maghrabi F, Alnajjar AA, Muzaffer M (2021). Clinical characteristics and outcomes of multisystem inflammatory syndrome in children (mis-C):A national multicenter cohort in Saudi Arabia. Curr Pediatr Res.

[11] Asseri AA, AlHelali I, Elbastawisi E, Ali AS, Al-Qahtani SM, Shati AA (2021). Multi-system inflammatory syndrome in children during the coronavirus disease 2019 in Saudi Arabia:Clinical perspective from a case series. Medicine (Baltimore).

[12] Henderson LA, Canna SW, Friedman KG, Gorelik M, Lapidus SK, Bassiri H (2020). American College of Rheumatology Clinical Guidance for Multisystem Inflammatory Syndrome in Children Associated With SARS-CoV-2 and Hyperinflammation in Pediatric COVID-19:Version 1. Arthritis Rheumatol.

